# Primary Cutaneous Nodular Amyloidosis Presenting As Violaceous Patches on Bilateral Plantar Feet

**DOI:** 10.7759/cureus.109556

**Published:** 2026-05-24

**Authors:** Madeline A LaRow, Paul K Vance, Michael Hohnadel

**Affiliations:** 1 Dermatology, Kirksville College of Osteopathic Medicine, Kirksville, USA; 2 Dermatology, Corpus Christi Medical Center, Corpus Christi, USA

**Keywords:** amyloidosis, apple-green birefringence, congo red staining, nodular amyloidosis, unusual acral dermatoses, violaceous patch

## Abstract

Primary localized cutaneous amyloidosis (PLCA) is characterized by amyloid deposition limited to the skin and is subdivided into macular, lichenoid, and nodular variants. Primary cutaneous nodular amyloidosis (PCNA) is the rarest subtype and typically presents as waxy, firm nodules or plaques composed of immunoglobulin light chain-derived (AL-type) amyloid. Plantar involvement is exceedingly uncommon. We report the case of a 64-year-old Hispanic man found incidentally to have bilateral, well-demarcated, flat violaceous patches on the plantar surfaces of both feet. The lesions were asymptomatic, non-nodular, and lacked overlying epidermal change. Histopathologic examination revealed amorphous eosinophilic deposits within the dermis, which stained positive with Congo red and exhibited apple-green birefringence under polarized light, confirming amyloid deposition. The patient was diagnosed with PCNA, presenting with atypical patch-like morphology, and managed conservatively with close clinical surveillance.

This case represents an unusual clinical presentation of PCNA, diverging from the classic nodular phenotype and mimicking inflammatory or granulomatous dermatoses. To our knowledge, this is among the first reported cases of plantar PCNA presenting as flat violaceous patches rather than nodules. Given the potential risk of progression to systemic amyloidosis, accurate diagnosis and ongoing monitoring remain essential. Awareness of this expanded clinical spectrum underscores the importance of histopathologic evaluation in atypical acral dermatoses and may help prevent misdiagnosis or delayed recognition of this rare entity.

## Introduction

Primary localized cutaneous amyloidosis (PLCA) represents a spectrum of disorders characterized by amyloid deposition confined to the skin, without evidence of systemic involvement. It is subclassified into macular, lichenoid, and nodular variants, with the nodular form (primary cutaneous nodular amyloidosis (PCNA)) being the rarest and most clinically distinctive [1]. It occurs in middle-aged adults and affects both sexes equally [2]. PCNA typically presents as asymptomatic, waxy, yellowish, or pink nodules or plaques, often located on the limbs, face, or trunk. Unlike the other types, these lesions are associated with dermal deposition of immunoglobulin light chain-derived amyloid (AL-type) and may rarely progress to systemic amyloidosis [3,4].

The plantar aspect of the foot is an exceptionally uncommon site for PCNA [5]. In the scant literature available, when PCNA does affect the soles, it almost invariably manifests as classic nodules or plaques. To date, there are only a few documented cases of PCNA occurring on the plantar foot, and all have typical nodular morphology [5-7].

In this report, we describe an unusual case of PCNA presenting as flat, violaceous patches on the bilateral plantar surfaces. This morphology is atypical for PCNA. This case expands the recognized clinical spectrum of PCNA while underscoring the importance of biopsy in evaluating acral dermatoses with unusual morphologies. It also emphasizes the diagnostic challenge of encountering an atypical presentation in the clinical setting.

## Case presentation

A 64-year-old Hispanic man presented to the clinic for an annual skin examination. On examination, bilateral erythematous to violaceous patches were noted on the plantar aspects of both feet. Palpation revealed a smooth surface with no palpable epidermal changes and very mild dermal induration. The patient reported no symptoms and was unaware of these lesions. The lesions were not present one year ago upon physical examination.

**Figure 1:**
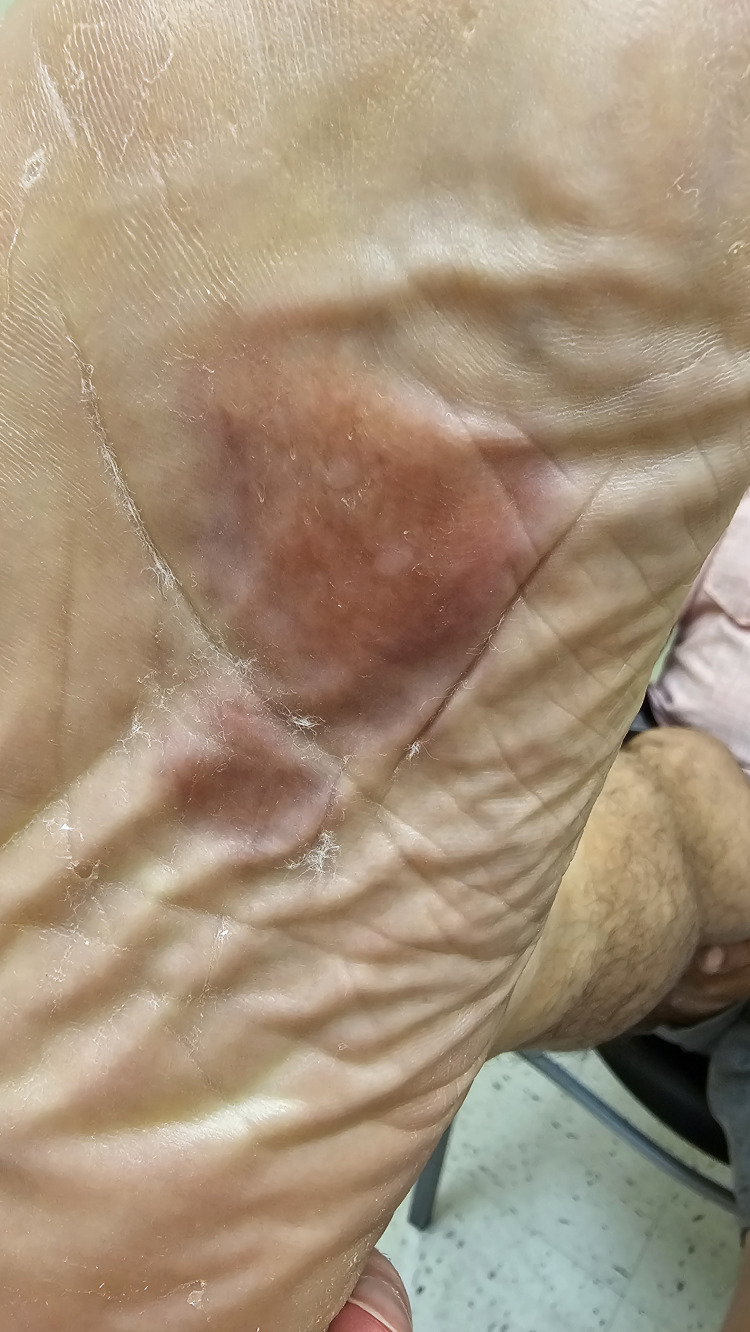
A 2.5 x 3.5 cm violaceous patch on the solar surface of the right foot

Physical examination revealed bilateral, discrete, well-demarcated, flat, violaceous patches 2.5 x 3.5 cm in diameter, distributed over the mid-plantar surface and medial arch of both feet (Figure 1). No nodularity, ulceration, or overlying hyperkeratosis was observed. The remainder of his cutaneous and mucosal examination was unremarkable.

Differential diagnoses considered at the time included necrobiosis lipoidica, vasculitis, morphea, ecchymosis, sarcoidosis, and fixed drug eruption. A 4-mm punch biopsy was obtained from one of the violaceous patches for histopathological evaluation.

**Figure 2:**
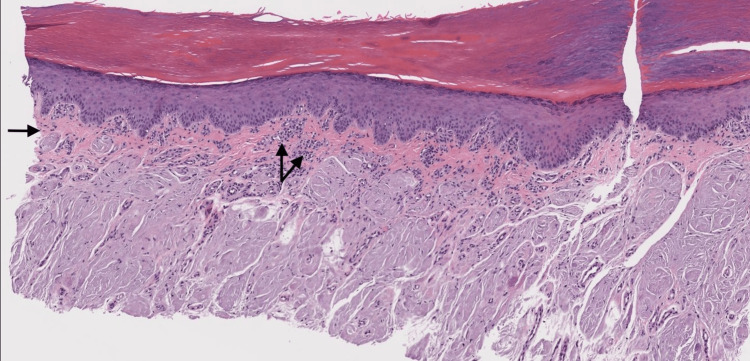
Hematoxylin and eosin stain (10x magnification) A dense homogenous band of eosinophilic amyloid deposits within the papillary and upper reticular dermis is shown with the left arrow. Superficial perivascular lymphoplasmacytic infiltrates are shown with a two-headed arrow.

Histologic examination revealed homogenous eosinophilic deposits within the papillary and upper reticular dermis, consistent with amyloid. There was a sparse superficial perivascular lymphoplasmacytic infiltrate (Figure 2). Congo red staining of the deposits confirmed amyloid, and under polarized light, the material exhibited classic apple-green birefringence. CK-AE1/3 and CK-HMW immunostains were negative.

To exclude systemic involvement, the patient underwent a complete systemic workup, including serum and urine protein electrophoresis, serum free light chain assay, renal and hepatic function tests, electrocardiogram (ECG), and echocardiogram. All results were within normal limits. There was no lymphadenopathy or organomegaly on physical examination or imaging.

The diagnosis of PCNA with atypical violaceous patch morphology localized to the plantar feet was established.

Given the asymptomatic and localized nature of the lesions, the patient was managed conservatively with regular clinical monitoring. At the three-month and nine-month follow-ups, the lesions remained stable in size and number, with no new lesions or systemic findings.

## Discussion

PCNA is the rarest subtype of PLCA, accounting for approximately 1.5% of all cutaneous amyloidosis cases [1]. Unlike the macular and lichenoid variants, which are associated with keratinocyte-derived amyloid, PCNA is histologically distinguished by dermal deposition of amyloid light chain (AL) protein, believed to originate from local plasma cell proliferation [3]. This molecular distinction is clinically important because PCNA, unlike other variants, may carry a 7% risk for progression to systemic amyloidosis, lower than the previously estimated rate of 50%. This process can occur years to decades after diagnosis despite the initial normal workup for systemic amyloidosis [2,4].

Clinically, PCNA most often presents as solitary or multiple waxy, firm, skin-colored to yellow-brown nodules or plaques anywhere on the skin, with a predilection for acral regions, such as the distal limbs, genitals, and head [2]. Violaceous patches on the bilateral plantar surfaces represent an unusual presentation. In their retrospective review of plantar dermatoses, Borrowman et al. reported a case of nodular amyloidosis on the anterior plantar foot initially misdiagnosed as a callus [6]. Similarly, Ferreira et al. described a PCNA lesion on a toe, but again with typical nodular morphology [8]. These examples underscore that even among rare plantar presentations, nodularity is the dominant pattern. 

Managing nodular amyloidosis can be difficult, as lesions frequently persist despite therapy or recur after initial improvement. Reported treatments include cryotherapy, surgical excision, pulsed-dye laser, intralesional or topical corticosteroids, and electrodesiccation with curettage. Systemic immunosuppression (e.g., cyclophosphamide) has been implemented in cases associated with underlying rheumatologic diseases, such as Sjögren syndrome and systemic sclerosis [9]. When counseling the patient and choosing a treatment modality, slow wound healing and a higher risk of poor scar formation should be considered, as amyloidosis can slow wound healing due to the presence of serum amyloid P [10].

The morphologically atypical flat, violaceous patches, in this case, mimic inflammatory and/or granulomatous dermatoses such as necrobiosis, vasculitis, and sarcoidosis. Clinically and dermoscopically, this could delay diagnosis and misguide clinicians. However, histology remains the cornerstone of diagnosis. In PCNA, hematoxylin and eosin staining reveals large, amorphous eosinophilic deposits in the dermis, often extending into the subcutaneous tissue. Congo red staining and polarization microscopy demonstrate the pathognomonic apple-green birefringence [8]. These features were observed clearly in our case, confirming the diagnosis despite the misleading clinical appearance.

## Conclusions

This case reinforces the importance of systemic evaluation, even in cases where lesions appear localized and asymptomatic. Although our patient has no evidence of systemic amyloidosis on laboratory and imaging studies to date, ongoing surveillance is warranted due to the known association between PCNA and progression to systemic diseases. Our case expands the clinical spectrum of PCNA, suggesting that non-nodular presentations, particularly on uncommon sites, such as the plantar foot, may be under-recognized. It is evident that PCNA does not always conform to its namesake nodular presentation. Therefore, histological confirmation remains invaluable for diagnosis. Broader recognition of this variability is essential for timely diagnosis and appropriate management.
